# CDK4: a master regulator of the cell cycle and its role in cancer

**DOI:** 10.18632/genesandcancer.221

**Published:** 2022-08-25

**Authors:** Stacey J. Baker, Poulikos I. Poulikakos, Hanna Y. Irie, Samir Parekh, E. Premkumar Reddy

**Affiliations:** ^1^Department of Oncological Sciences, Icahn School of Medicine at Mount Sinai, Levy Place, NY 10029, USA; ^2^Tisch Cancer Institute, Icahn School of Medicine at Mount Sinai, Levy Place, NY 10029, USA; ^3^Department of Hematology and Medical Oncology, Icahn School of Medicine at Mount Sinai, Levy Place, NY 10029, USA; ^4^Department of Pharmacological Sciences, Icahn School of Medicine at Mount Sinai, Levy Place, NY 10029, USA

**Keywords:** CDK4/6, cancer, cell cycle, targeted therapy, checkpoint inhibitor

## Abstract

The cell cycle is regulated in part by cyclins and their associated serine/threonine cyclin-dependent kinases, or CDKs. CDK4, in conjunction with the D-type cyclins, mediates progression through the G_1_ phase when the cell prepares to initiate DNA synthesis. Although *Cdk*4-null mutant mice are viable and cell proliferation is not significantly affected *in vitro* due to compensatory roles played by other CDKs, this gene plays a key role in mammalian development and cancer. This review discusses the role that CDK4 plays in cell cycle control, normal development and tumorigenesis as well as the current status and utility of approved small molecule CDK4/6 inhibitors that are currently being used as cancer therapeutics.

## INTRODUCTION TO THE CELL CYCLE

The mammalian cell cycle is divided into four phases, Gap 1 (G_1_), Synthesis (S), Gap 2 (G_2_) and Mitosis (M), whose order and timing are critical for accurate transmission of genetic information. Consequently, a number of biochemical pathways have evolved to ensure that initiation of a particular cell cycle event is dependent on the accurate completion of another. These biochemical pathways have been termed “checkpoints” [reviewed in 1].

One of the major breakthroughs in our understanding of cell cycle regulation was the discovery of the *cdc2+* and *cdc28* genes in *Schizosaccharomyces pombe* and *Saccharomyces cerevisiae*, respectively. Both genes encode two related kinases, termed cyclin dependent kinases or CDKs, and their activities are required during the G_1_/S and G_2_/M transitions. While a single a CDK triggers the major transitions of the yeast cell division cycle, mammalian cells encode multiple *CDC2*-related genes [reviewed in 2]. The discovery of more than 10 different CDC2-related proteins in vertebrates initially led to speculation that regulation of the cell cycle in higher eukaryotes might involve a complex combination of CDKs and cyclins. However, subsequent studies have shown that the majority of these CDKs are not critical regulators of the cell cycle.

CDKs are holoenzymes composed of a regulatory subunit, called a cyclin, and a catalytic subunit, termed a cyclin-dependent kinase [reviewed in 1, 3, 4]. CDKs are serine/threonine kinases that have catalytic domains of roughly 300 amino acids and are inactive when underphosphorylated and monomeric [reviewed in 3]. The primary mechanism of CDK activation is the association with regulatory cyclin partners. Unlike CDKs which are highly homologous, cyclins are a remarkably diverse family of proteins, ranging in size from approximately 35–90 kDa [reviewed in 1, 3, 5]. Sequence homology amongst the cyclins tends to be concentrated in a 100 amino acid domain known as the cyclin box, which is necessary for CDK binding and activation. Complete activation of most CDKs also requires phosphorylation of a conserved threonine (Thr) residue located in the T-loop by CAK1/CDK7, a cyclin-dependent kinase that has been shown to phosphorylate the catalytic subunit of various CDKs, including the residue that is equivalent to Thr^161^ of CDC2, which activates the kinase activity of their holoenzymes. In CDK4, this phosphorylation occurs at Thr^172^ [6]. Interestingly, the activity of this protein complex does not change in a cell cycle-dependent manner and is present even in quiescent cells [7].

In mammalian cells, cell cycle progression requires a series of events that culminate in the expression and assembly of different CDKs [reviewed in 1, 3, 5]. CDK4/6 associate with D-type cyclins and mediate progression through the G_1_ phase when the cell prepares to initiate DNA synthesis. Activation of CDK4/6/CYCLIN D complexes contributes to hyperphosphorylation of the retinoblastoma (RB) protein and its related proteins, p107 and p130. The hypophosphorylated form of pRB binds to and sequesters several cellular proteins, and its phosphorylation results in the release of these protein factors. One key binding partner is the transcription factor E2F-1, which appears to positively activate the transcription of genes whose products are required for S-phase progression. E2F-1 and other members of the E2F family are known to bind to pRB and heterodimerize with DP-1 and -2, an interaction that is required for the DNA-binding capacity of E2F family proteins [reviewed in 1, 3–5, 8, 9]. Once the cell has made the G_1_/S transition, CYCLIN E/CDK2 phosphorylates the remaining residues on the RB family proteins that are critical for E2F activation. Activation of E2F-mediated transcription allows the cell to transit into S phase and initiate DNA replication, which is controlled, in part, through CYCLIN A/CDK2. CYCLIN A/CDK2 ultimately forces the cell through the G_2_ phase prior to the assembly of the CYCLIN B/CDK1 and the initiation of mitosis [reviewed in 9].

## REGULATION OF CDK4 ACTIVITY

A key response to many growth factors in many cell types is the activation of CDK4 or CDK6 by members of the CYCLIN D family (D1, D2 and D3). Although D-type cyclins are absent in quiescent cells, they are important integrators of mitogenic signaling. CYCLIN D expression is stimulated by and is dependent on growth factors, and consequently, if these factors are removed, CYCLIN D levels drop immediately regardless of the stage of the cell cycle. A fully active CDK/CYCLIN complex can be turned off by at least two different mechanisms. Regulatory kinases can phosphorylate the CDK subunit at inhibitory sites near the N-terminus, or, CYCLIN/CDK complexes can be negatively controlled in a tissue-restricted manner by 2 families of cyclin kinase inhibitors (CKIs), the INK4 and CIP/KIP families of proteins [reviewed in 1, 10–14].

**Figure 1:**
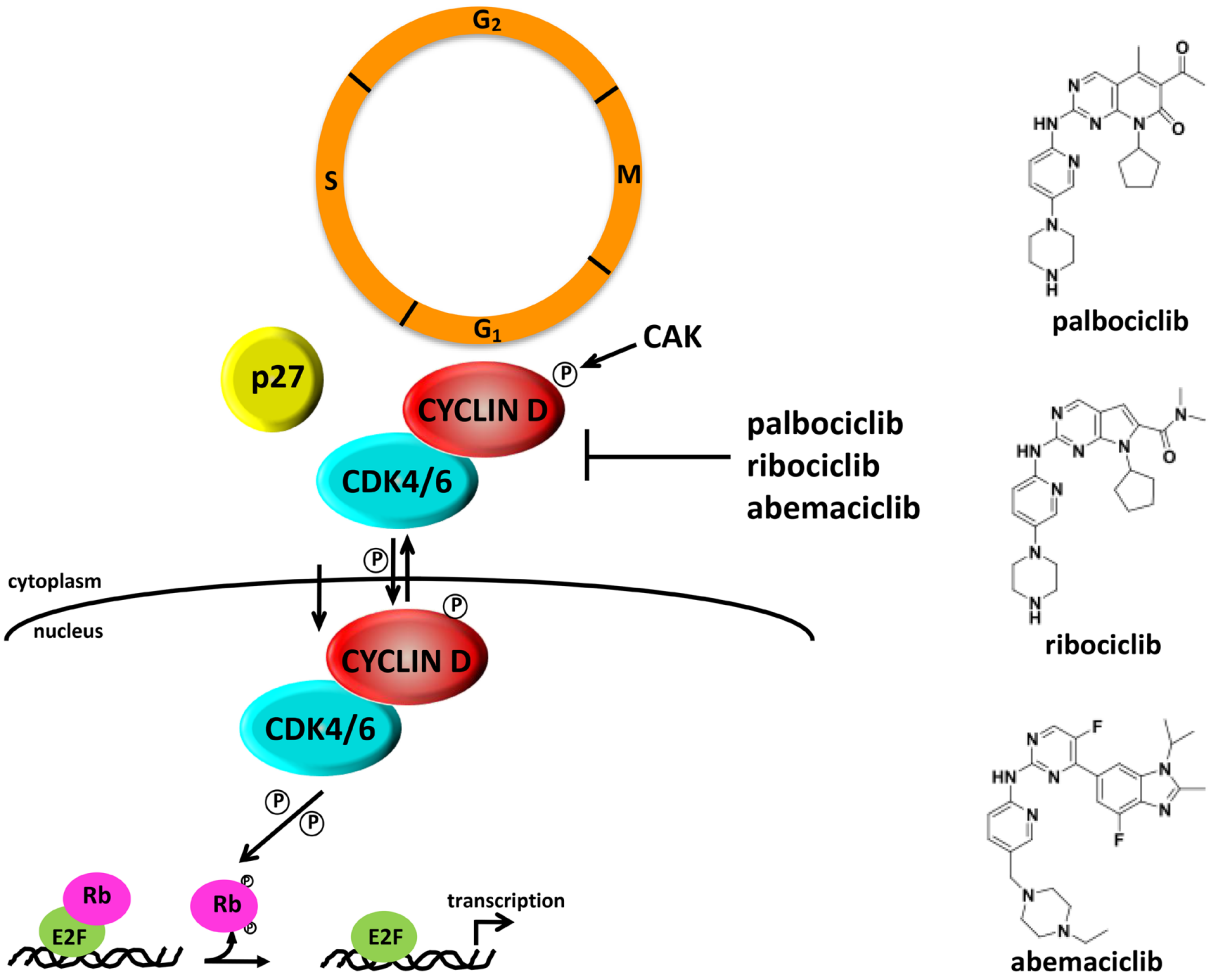
CDK4 regulation, activation and inhibition during cell cycle progression. Structures of CDK4/6 specific small molecule inhibitors are shown on the right. (Adapted and modified from [50]).

The INK4 family of proteins (p16^INK4A^, p15^INK4B^, p18^INK4C^, p19^INK4D^) inhibit D-type cyclin activity by specifically associating with CDK4 and CDK6 (Figure 1). These inhibitory proteins are expressed at low or undetectable levels in proliferating cells and are rapidly induced by growth inhibitory stimuli, such as contact inhibition, senescence, or treatment with certain growth inhibitory factors [reviewed in 11]. Of the four INK4 proteins, p16^INK4A^ seems to play a critical role in senescence and tumor suppression in human cells [reviewed in 10, 13]. p16 is exclusively composed of four ankyrin repeat motifs, which are relatively well conserved motifs of 31-34 amino acids that mediate protein-protein interactions. In solution, the four ankyrin repeats are stacked together in a linear fashion to form a helix bundle with a concave surface which harbors clusters of charged groups that mediate protein-protein binding [reviewed in 14, 15]. The crystal structure of the p16-CDK6 complex has been solved [reviewed in 14, 15], and these studies show that binding of CDK6 to the charged domain of p16 results in an electrostatic interaction between Asp^84^ of p16 and Arg^31^ of CDK6 (which corresponds to Arg^24^ in CDK4). Because these residues are located in the active site of these two CDKs, this interaction diminishes kinase activity. In addition, this interaction appears to impair the binding of CDK4 and CDK6 to CYCLIN D, as it “shrinks” the CYCLIN D binding surface. This is consistent with our observation that oncogenic mutations at the Arg^24^ residue of CDK4 results in an inhibition of p16 binding, which in turn results in enhanced kinase activity and increased cell proliferation [16, 17].

The CIP/KIP family (p21^CIP1^, p27^KIP1^, p57^KIP2^) of proteins binds and inactivates CDK2/CYCLIN E, CDK2/CYCLIN A and CDK1/CYCLIN B complexes. Structure/function analysis of the p21 and p27 proteins show that their N-termini contain two key domains, one that is required for cyclin binding and another that is required for association with the CDK subunit. The cyclin binding motif appears to be important for providing high-affinity binding and is believed to underlie the specificity of CIP/KIP proteins for all cyclin-containing complexes [18, 19, reviewed in 12, 20, 21]. p27 is actually expressed throughout the cell cycle and a majority of this protein in proliferating cells is thought to be associated with CYCLIN D-CDK4 complexes. These p27-CYCLIN D-CDK4 complexes possess kinase activity, suggesting that this interaction does not result in an inhibition of CDK4 [22–30; reviewed in 11, 12]. In this case, p27 appears to stabilize CYCLIN D-CDK4 complexes, as increased expression of p27 has been shown to result in increased CDK4 kinase activity. This observation was confirmed using p27−/− mouse embryo fibroblasts (MEFs), whereby CDK4 enzymatic activity is reduced [30, 31]. Together, these studies suggest that CYCLIN D-CDK4/6 complexes exhibit a non-catalytic function, whereby their association with p21 and p27 in the G_1_ phase sequesters these CKIs and prevents their binding to CYCLIN E/CDK2 to allow progression through G_1_.

Both p21 and p27 have also been shown to inhibit the CYCLIN D/CDK4/6 complex under certain growth conditions [32–34]. p27 levels increase dramatically in response to certain anti-proliferative signals and under these conditions, cyclin D-CDK4 complexes are inactive [22, 23: reviewed in 11, 12]. These observations suggest that p27 could act both as an inhibitor and activator of CDK4-CYCLIN D complexes depending on cellular context (Figure 1). James et al. [25] reported that p27 is preferentially tyrosine phosphorylated at positions 88 and 89 in proliferating cells, causing it to bind CYCLIN D-CDK4 complexes in a non-inhibitory fashion. Treatment of tyrosine-phosphorylated p27 protein preparations with a phosphatase converted the p27 molecule to its inhibitory form, suggesting that p27 functions as an important molecular switch that discerns between growth inhibitory and growth promoting signals. The significance of the binding of p27 to CYCLIN D/CDK4 complexes and how it dictates response to targeted CDK4 therapies will be discussed later in this article.

## CDK4 TARGETS

By far, the most studied G_1_ CYCLIN/CDK substrates is RB, which is phosphorylated in a cell cycle dependent manner. RB is hypophosphorylated in quiescent cells and becomes phosphorylated on Ser^780^ and Ser^795^ by CDK4/CDK6 during mid to late G_1_. The hypophosphorylated form of pRB associates with several cellular proteins, and its phosphorylation results in the disassociation of RB from its binding partners [35–37]. One such protein is the transcription factor E2F-1, which activates the transcription of genes whose products are required for S-phase progression. Most of the E2F-responsive genes identified so far are required for the G_1_ transition to the S phase of the cell cycle, being transcriptionally activated in a period of G_1_ that coincides with passage through the restriction point. The two other RB-related genes that encode pocket proteins with similar biochemical activity, p107 and p130, are also substrates of CYCLIN/CDK complexes. For example, p107 is phosphorylated by CYCLIN D/CDK4/6 on Ser^842^ [37]. Studies have shown that hypophosphorylated RB preferentially associates with certain histone deacetylases (HDACs) [38–40]. According to this model of RB-mediated chromatin repression, the RB-E2F1-HDAC complex binds to the promoters of S-phase specific genes, where the HDAC acts on surrounding chromatin and causes it to adopt a closed conformation. Phosphorylation of RB by CDK4/6 appears to result in the dissociation of the repressor complex, which in turn allows the expression of CYCLIN E [reviewed in 41]. CYCLIN D-CDK4/6-mediated phosphorylation of RB not only permits dissociation of the HDACs, but also appears to result in the recruitment of the CYCLIN E-CDK2 complex to the RB pocket. Under these conditions, the hypoacetylated state of chromatin is no longer maintained and histone acetylation results in the opening of chromatin structure and the activation of transcription [38–40].

In addition to these proteins, other CDK4 substrates include, but are not limited to, SMAD2/3, CDT1 and MARCKS, FOXM1, PRMT5, and several of these proteins have been shown to serve as substrates for other CDKs as well [42–46]. Interestingly, CDK4 does not phosphorylate p27 or histone H1, a canonical CDK substrate [22, 47–49], and when compared to other CDKs, the number of bona fide CDK4 substrates is relatively small [reviewed in 50]. Crystal structures of CDK4/Cyclin D complexes suggest that the active conformation of CDK4 is highly dependent on binding to both substrate and cyclin [51].

## ROLE OF CDK4 IN MAMMALIAN DEVELOPMENT AND CANCER AS REVEALED BY GENETICALLY MODIFIED MOUSE MODELS

### Knock-out mouse models of CDKs

As transition through each phase of the cell cycle is dependent on sequential activation of multiple CDKs, it was believed that unless there are compensatory effects by another CDK that is co-expressed (as seen with CDK4 and CDK6), loss of a single CDK would have detrimental effects on development or cell cycle progression. Such is the case of CDK1, whereby loss of CDK1 expression results in embryonic lethality at the blastocyst stage of development [52]. CDK1 is actually sufficient to drive mitosis in the absence of any interphase CDKs and can restore meiosis in oocytes, owing to its ability to bind to all cyclins and phosphorylate RB [53, 54]. Mice lacking either CDK2, 3, 4 or 6 are viable and cell proliferation is not significantly affected *in vitro* due to compensatory roles played by other CDKs [16, 55–59]. Nevertheless, these studies do not preclude a role for individual CDKs in mammalian development and disease. Even though *Cdk2/Cdk4*-null MEFs display normal S-phase progression, they eventually become immortalized and express high levels of phosphorylated RB [60]. However, knock-out of these genes in the mouse results in lethality that is likely caused by cardiac failure, a phenotype that is similar to *Cyclin D1*, *D2* and *D3* triple-knockout mice. [60, 61]. Similarly, MEFs isolated from *Cdk4/6* double knock-out embryos proliferate *in vitro* with only slight defects in S phase, yet the embryos die *in utero* due to anemia [61]. Even though it was assumed that CDK4 and CDK6 have compensatory roles, knock-out of each of these loci individually has revealed unique roles for both proteins. This is not surprising given that their patterns of expression do not overlap completely. Systemic loss of *Cdk6* in mice only results in a slight impairment of the mature cells that comprise the lymphoid tissues, although recent studies with conditional mouse models show a definitive role in thymocyte development, proliferation and transformation [62, 63]. The phenotype of *Cdk4* null-mutant mice is actually quite different.

### CDK4-knockout mice

Mice that are nullizygous for the *Cdk4* allele exhibit a diabetic-like phenotype, with a 90% reduction in glucose levels, polyuria, polydipsia and dramatic reductions in the size and number of pancreatic ß-islet cells [16]. Both male and female mice are infertile, with males exhibiting testicular atrophy due to meiotic abnormalities and embryos failing to undergo implantation in females that otherwise ovulate normally [16, 64]. Females also display pituitary hypoplasia that is characterized by a reduction in the number of prolactin-producing lacotrophic cells [16, 64–67]. Interestingly, *Cdk4*-null animals are smaller in size compared to their wild-type littermates and it is therefore not surprising that both the size and number of both ß-islets and lacotrophs are also reduced in these animals. Although genetic rescue restores proliferation of both cell types (and thereby corrects the defects in glucose levels and female fertility) the number of these specialized cells remains reduced [68]. These data demonstrate that the observed phenotypes are the result of reduced cycling of β-cells, leading to a loss of β-islets as opposed to defects in the functionality of these cell types. That CDK4 plays a key role in homeostasis and cell cycle entry is also revealed through cell cycle experiments using serum-starved CDK4-deficient MEFs that exhibit a considerable delay in reaching S-phase and remain in G_1_ for prolonged periods of time [16, 17, 58]. In addition to these overt phenotypes, *Cdk4* null-mutant mice are also prone to neurological defects such as impaired locomotion, staggering and hyperactivity, have abnormalities in thymocyte maturation and allergen response, and exhibit impaired adipocyte differentiation and function [16, 69, 70; reviewed in 50].

While *Cdk4* null-mutant mice underscore a role for the gene in normal cell development, this animal model has also shed light on the role that this kinase plays in the genesis and progression of cancer, particularly that of the mammary gland. As discussed above, a key response to growth factors in many cell types is the activation of CDK4 or CDK6 by D-type CYCLINS. Approximately 50% of human mammary carcinomas express abnormally high levels of CYCLIN D1 [71–75] that are maintained throughout subsequent stages of breast cancer progression from *in situ* carcinoma to invasive carcinomas [74, 76, 77]. *CDK4* is also amplified or overexpressed in a variety of tumor types, including sarcomas, gliomas, lymphomas and those of the breast [reviewed in 1].

The absence of CDK4 expression in the mammary gland results in defective mammary gland development, with the mammary glands of virgin female *Cdk4*-null mice displaying defects in ductal outgrowth, a reduction in the number of mammary ducts and a complete absence of alveoli [78]. Expression of the MMTV-driven *Neu* oncogene in wild-type animals results in the appearance of infiltrating hyperplastic and dysplastic nodules in the mammary gland that is considerably reduced in the absence of CDK4 expression. *Cdk4*-null females also fail to show any of the proliferative disturbances that are otherwise normally observed as a result of *Neu* expression. As a result, the onset and incidence of mammary carcinoma in MMTV-*Neu-Cdk4*−/− mice are delayed and substantially reduced, respectively. Interestingly, loss of CDK4 expression does not affect the onset or incidence of mammary tumors that result from WNT-1 expression. Although it has been reported that CYCLIN D2 expression compensates for loss of CYCLIN D1 expression in *Wnt*-driven mammary tumors [79], neither CDK2 nor CDK6 appear to compensate for the absence of CDK4 expression [78]. It has also been suggested that WNT and c-MYC communicate with the cell cycle machinery in breast epithelial cells through different targets during tumorigenesis in the mammary gland. In this regard, CYCLIN D1 expression is up-regulated in tumors induced by *Wnt-1* and *c-Myc*, but not by *Neu* or *Ras* [79].

Studies on MMTV*-Neu/p16* double-transgenic mice show that *Neu*-mediated tumorigenesis is blocked by *p16* and that these double-transgenic mice develop rare tumors after a long latency [80]. Because MMTV-*Neu*-*Cdk4*−/− mice showed decreased levels of ductal branching and lobuloalveolar development of the mammary glands when compared to control animals, it is presumed that CDK4 is required for these proliferative events that are induced by *Neu*. These studies do not rule out the possibility that the observed defects in *cdk4*-null mammary gland development could be an indirect result of hormonal signaling deficiencies as opposed to an epithelial cell autonomous defect. Therefore, if the defect in mammary development observed in *cdk4*-null females is not cell autonomous, then WNT not only bypasses CDK4 function, but also any conceivable defects in hormone signaling resulting from *Cdk4* ablation. Considering previous results indicating that *Neu* acts by inducing CYCLIN D1 expression, and the fact that that *Cdk4* is required for *Neu-*induced tumorigenesis, it is probable that the CYCLIN D1/CDK4 complex itself is required for *Neu*-induced tumorigenesis. This is highly likely as mammary gland development in knock-in mice expressing a kinase-defective CYCLIN D1 mutant that does not associate with CDK4/6 complexes proceeds normally, and yet, these animals are resistant to *Neu*-induced tumorigenesis [81, 82]. However, it is important to remember that the oncogenic function of CYCLIN D1 may be partly independent of its ability to activate CDKs and is perhaps linked to the direct effects of CYCLIN D1 in controlling the expression of a subset of genes that are co-up-regulated in human tumors with deregulated *CCND1* [83]. In spite of the fact that the mechanism is not fully defined, the lessons learned from *Cyclin D*1 and *Cdk4* null mouse models have important implications with respect to therapeutic modalities that might be effective in the treatment of breast cancers that are HER2-positive.

Although *cdk4*-null-mutant mice highlight the importance of the CDK4/CYCLIN D1 complex in breast tumors and provide evidence to suggest that small molecule inhibitors of CDK4 kinase activity could be effective in the treatment of human disease, the importance of mutations in the *CDK4* locus in human cancer was first underscored by discoveries which showed that germline mutations in this gene which abolish the ability of the encoded protein to bind to p16^INK4A^ result in a predisposition of individuals to the development of melanoma [84, 85]. The CDK4*-*Arg24Cys (R24C) mutation was also detected in sporadic melanomas [84], suggesting that a *CDK4* gene containing this mutation could act as a dominant oncogene that is resistant to normal physiological inhibition by p16^INK4A^.

### CDK4^R24C^ knock-in mice

Because the R24C mutation abolishes the ability of CDK4 to interact with p16, it was thought that the phenotype of both the *cdk*^R24C^ mutant mice and the *p16* null mutant mice would be identical. However, this is not the case [16, 17, 86] and their phenotype more closely resembles that of *p16/p19* double knock-out animals [87]. Although CDK4^R24C^ mice develop a variety of spontaneous primary and metastatic tumors [16, 17, 86], the major pathological abnormality observed in these animals is the onset of pancreatic islet cell hyperplasia during the first three months of life. Islets are primarily comprised of insulin-secreting ß cells and interestingly, as stated above, mice that are null for CDK4 expression develop insulin-deficient diabetes. Together, both models illustrate a critical and highly specific role for *Cdk4* in the development and proliferation of this particular cell type. As expected, the CDK4^R24C^ protein isolated from MEFs does not associate with p16^INK4A^ and is therefore not subject to its negative regulatory effects as is evidenced by the increased expression of hyper-phosphorylated members of the RB family. CDK4^R24C^–expressing MEFs exhibit decreased doubling times, with a slightly higher percentage of cells in the S and G_2_M phases, and fail to undergo senescence. The fact that long-term cultured cells (20 passages or more) spontaneously form foci, and that the MEFs themselves are highly susceptible to Ha-*ras,* *E1A* and v-*myc* oncogene-driven transformation, suggests that the *cdk4*^R24C^ mutation serves as a primary event in the progression towards a fully transformed phenotype [16, 17]. CDK4^R24C^ mice are also susceptible to an increase in the development of pituitary tumors arising either in the pars intermedia or the pars distalis with characteristic angiomatous areas or dilated “blood-filled lakes” of various sizes. In many cases, the pituitary tumors compressed adjacent non-tumorous tissues, such as the hypothalamus and pons. Interestingly, mice that are heterozygous at the *Rb* loci and those that have disruptions in *p27*^Kip1^ and *p18*^Ink4c^ also develop pituitary tumors [88–93], and it has been shown that overexpression of the high mobility group AT-hook protein 2 (HMGA2) cooperates with loss of p27 expression or expression of CDK4^R24C^ to promote pituitary tumor development and progression [94]. While the germline R24C mutation predisposes humans to hereditary melanoma, in general, there is a low level of spontaneous melanoma occurrence in CDK4^R24C^ mice [17, 95–97]. This observation suggests that other mutagenic events, such as exposure to UV radiation or other carcinogens could play a major role in this process, and is consistent with reports demonstrating that melanoma development in *p16/p19* double knock-out mice is dependent on the expression of the *H-Ras*^G12V^ transgene [97].

Studies using the two-step model of skin carcinogenesis that involves sequential treatment with the mutagen 9, 10-di-methyl-1,2-benz[*a*]anthracene (DMBA) and tumor promoter 12-*O*-tetradecanoylphorbol-13-acetate (TPA) and the effects on skin in *Cdk4*^R24C^ mice have also been reported, but with slightly different results [86, 95–97]. We have shown that *Cdk4*^R24C^ heterozygous and homozygous mice form papillomas with regions of hyperplasia in the epidermis with a very short latency period [96]. No invasion into the underlying dermis was observed and there was also a reduced incidence of benign epidermal tumors (classified as keratocanthomas), consisting of large keratin-filled cystic structures surrounded by a very well differentiated squamous epithelium [17, 96]. These results differed from what was reported by Sotillo et al. [95], who treated the animals with the same carcinogen and tumor promoter, but showed that the mice developed both papillomas and melanomas. The main differences between these two studies were the age at which the mice were treated and the dose of the DMBA/TPA. Mice that received larger doses of TPA starting at a younger age also developed melanomas. In general, treatment with DMBA/TPA results in the development of papillomas at the site of initiation and promotion with a characteristic oncogenic mutation in the 61st codon of the *Ha*-*ras* gene [98, 99] and examination of the skin papillomas in the DMBA/TPA treated mice contained this mutation. However, as only 10% of the melanomas contained mutations in the *H-ras* and *N*-*ras* genes in the Sotillo et al. study [95], these results suggest that other genes are targets of DMBA in these animals and/or that *Ras* is not necessarily the gene that “drives” melanoma initiation in the response to the R24C mutation, at least in response to DMBA/TPA.

Nevertheless, these studies do not preclude a role for *Ras* genes in CDK4^R24C^-mediated melanoma development. Several studies using tyrosinase-*Hra*s (Tyr-*HRas*)/*cdk*^R24C^ mice report that compound mice develop melanomas in response to DMBA/TPA and UV radiation [95–97]. These mice also develop spontaneous melanomas, albeit at a lesser frequency, and all spontaneous melanomas tested in our study showed activation of the RAS pathway [96]. While these results indicate that additional changes at the genetic level are required for maximal penetrance and tumor incidence, CDK4-deficiency in mice inhibits the development of DMBA/TPA-induced skin tumors even though the proliferation of keratinocytes and wound healing proceed normally in these animals. In normal keratinocytes, CDK6, and to a lesser extent CDK2, appear to compensate for the loss of CDK4 activity [100]. It is therefore likely that in the case of CDK4, the R24C mutation contributes to tumor progression and aggressiveness in melanomas that are initiated by H-*ras* activation or other changes in gene expression [101–103]. This is also likely the case with other tumor types, such as those that arise in the colons of *apc*^+/Min^*cdk4*^R24C^ mice [104].

In addition to pancreatic, thyroid and skin tumors, CDK4^R24C^ female mice develop severe mammary duct dilation and a high incidence and burden of aggressive mammary tumors [17]. The majority of the mammary tumors analyzed were adenosquamous carcinomas with papillary and cribiform elements or adenocarcinomas and adenocanthomas with squamous differentiation. The tumor cells lining the cavities undergo squamous differentiation or metaplasia with keratinization and formation of laminated horny pearls. This is consistent with the observation that MMTV-*cyclin D1* transgenic mice are prone to a high frequency of adenocarcinomas and adenocanthomas [105]. As *Cdk4* is required for *vHa-ras*-driven mammary tumorigenesis [106], and given that CDK4^R24C^ females have an increased incidence of spontaneous mammary tumors and that the CDK4^R24C^ protein cooperates with RAS to drive melanoma formation, it is surprising that co-expression of CDK4^R24C^ and v*Ha-ras* in mammary epithelial cells delays the onset of tumorigenesis. This is not due to reduced proliferation as both control and CDK4^R24C^-expressing tumors express comparable levels of proliferative markers. Rather, expression of RAS and CDK4^R24C^ leads to activation of senescence pathways and induction of apoptotic and DNA damage pathways [106]. Loss of CDK4 expression also results in the senescence of pre-neoplastic cells in the lung and blocks the development of lung tumors in mouse models [107]. Although the *KRAS-G12V* transgene is expressed in multiple tissues, tumor induction and subsequent senescence due to an absence of CDK4 expression were only detected in the lung. It is at present unclear as to why only cells of the lung are dramatically affected. These observations are consistent with the RAS isoform and codon mutation biases that are typically present in malignancies of the lung [reviewed in 108], although it is possible that there are additional changes in gene expression that occur only in lung tumor tissue. Given that oncogenic mutations can result in a tumor cell’s dependence on CDK4, and that CDK4 regulates breast cancer tumor cell stemness [109], these studies suggest that the development of CDK4-specific inhibitors may be beneficial in the treatment of cancer types that rely predominantly on CDK4 expression.

## CDK4 AND ITS ROLE IN NORMAL AND TUMOR CELL METABOLISM

While cell cycle regulatory proteins such as Rb, CYCLIN D3 and CDK9, regulate lipid and oxidative metabolism in adipocytes [110–112, reviewed in 113], the possibility that CDK4 itself might play a role in metabolism arose from the observations that *Cdk4*-null mutant mice develop a diabetic-like phenotype [22] and that disruption *Cdk4* alleles or expression of those that encode the R24C activating mutations in primary MEFs results in reduced and increased adipogenic potential, respectively [69]. Furthermore, adipocytes isolated from *Cdk4*^−/−^ mice exhibit decreased expression of genes that play a role in lipogenesis, decreased insulin sensitivity and glucose uptake [69]. CDK4 has since been shown to be an essential mediator of insulin response in adipocytes [114].

Studies investigating the role that CDK4 plays in glucose metabolism revealed that in mice, the role that CDK4 plays in glucose metabolism is independent of its cell cycle regulatory activities. Insulin activates CYCLIN D1-CDK4 complexes which suppress glucose production in the liver via the GCN5 (general control non-repressed protein 5) histone acetyltransferase [115]. Amino acids in the diet actually increase the level of *Ccnd1* mRNA and CYCLIND1/CDK4 complexes activate GCN5, resulting in an inhibition of gene expression of genes that regulate gluconeogenesis. In agreement with these studies, levels of CYCLIND1/CDK4 complexes are increased in mouse models of diabetes and remain at an increased steady state level, even in response to changes in food intake and/or fasting [115].

In addition to glucose metabolism, CDK4 and CYCLIN D3 also plays a role anaerobic glycolysis and fatty acid oxidation (FAO) via modulation of AMPK (AMP-activated protein kinase) activity [116]. In this setting, CDK4 negatively regulates FAO via phosphorylation of the AMPKα2 subunit. While *Cdk4* null mutant MEFs phenocopy cells that have been treated with an AMPK activator and display high levels of FAO and low levels anaerobic glycolytic activity, cells expressing the activating CDK4^R24C^ mutant protein exhibit the opposite phenotypes. Because metabolic pathways are often subverted in tumor cells, the roles that CDK4 and CDK6 play in malignant cell metabolism as it relates to cell survival is an active area of research. For example, studies have shown that in tumors that express high levels of CYCLIN D3/CDK6 complexes, they simultaneously regulate cell cycle progression and survival, in addition to directring a metabolic shift to the the pentose phosphate shunt. Interestingly, tumor types that express other forms of CYCLIN D-CDK4/6 complexes, such as breast cancers in which CDK4 is often in complex with CYCLIN D1, do not undergo apoptosis or exhibit changes in the phosphorylation status of metabolic enzynes in response to CDK4 inhibition [117]. The role that CDK4 plays in cancer and the development and utility of CDK4/6-targeted therapies in various tumor types are discussed in greater detail in the remainder of this article.

## THE ROLE OF CDK4 IN HUMAN CANCER

It is now believed that a vast number of human tumors exhibit deregulation of the CDK4/6-CYCLIN D-INK4-RB pathway by multiple mechanisms. For example, CDK4/6 is hyperactivated in many human cancers as a result of overexpression of positive regulators such as CYCLIN D, inactivation of INK4 and CIP/KIP inhibitors, or deletion and/or epigenetic alterations of substrates such as RB [reviewed in 1, 118, 119]. Hyperactive CDK4 has been reported in epithelial malignancies in the endocrine tissues and mucosa while CDK6 activation was reported in certain mesenchymal tumors such as sarcomas and leukemias [reviewed in 1]. Mutations and chromosomal translocations in the *CDK4* and *CDK6* loci have also been described. One of the best examples is the CDK4^R24C^ mutation that results in insensitivity to INK4 family inhibitors and was first described in patients with familial melanoma [84, 85]. An analogous point mutation in *Cdk6* that blocks the interaction of p16INK4a with CDK6 has also been reported in a human neuroblastoma cell line [120]. Chromosomal translocations within the *CDK6* promoter that lead to CDK6 overexpression were also described in splenic marginal zone lymphomas and B-cell lymphocytic leukemias [121, 122]. Finally, *CDK4/6* amplification or overexpression has also been observed in a wide spectrum of tumors, including gliomas, sarcomas, lymphomas, melanomas, cancers of the breast, squamous cell carcinomas and leukemias [reviewed in 118, 123].

CDK4 expression is also a prognostic indicator in certain cancers, such as triple negative breast cancer (TNBC). Studies have shown that CDK4 is highly expressed in this tumor type and correlates with poor survival and gene signatures that are associated with metastasis [109]. One of the primary reasons for TNBC recurrence is thought to be due to the presence of disproportionately large numbers breast cancer stem cells (BCSCs) within these tumors. These cells are a sub-population of cells which are resistant to standard chemotherapeutic agents, are long-lived, have the ability to self-renew, grow as mammospheres *in vitro* and initiate tumor development in mouse models [124–127]. CDK4 is a regulator of TNBC BCSC self-renewal and does so, in part, by down-regulating the expression of bone morphogenic protein-4 (BMP-4) [109], a key player in tumor stem cell biology [128]. Importantly, CDK4 also promotes the self-renewal and proliferation of chemotherapy-resistant TNBC BCSCs, which are largely responsible for the recurrence and metastasis in this aggressive cancer [109]. Given that inhibition of CDK4 activity in TNBC cell lines results in BCSC differentiation [129] and loss of self-renewal [109], blocking CDK4 activity in this and other tumor types is a sound approach and has resulted in the approval of several CDK4/6 ATP-mimetics for use in the clinic.

## TARGETING CDK4 FOR CANCER THERAPY

### Approved small molecule inhibitors of CDK4/6

Early evidence that CYCLIN D and CDK4/6 activities are upregulated in certain tumor cell types, and that *cdk4-/-* mice fail to develop *MMTV-neu* and *MMTV-ras*-induced mammary tumors, led to concerted efforts to develop small molecule inhibitors for these kinases. The first generation of CDK inhibitors developed, e.g., flavopiridol and roscovitine, potently inhibited CDK4 but were non-selective, inhibited multiple kinases and caused severe toxic side effects when these molecules entered clinical trials [reviewed in 21, 130].

In an attempt to overcome the toxicity profile of pan-CDK inhibitors, several groups undertook the initial development of next generation CDK inhibitors that are specific for individual CDKs. Some of these compounds exhibited a high degree of selectivity towards CDK4/6 by targeting the ATP binding site of CDK4/6-CYCLIN D complexes. Structures and IC_50_ values of potent CDK4/6 selective compounds, members of chemical classes of oxindoles, triaminopyrimidines, diarylureas, thioacridones, benzothiadiazines, indolocarbazoles, and pyrido[2,3-*d*]pyrimidines, have been summarized elsewhere [reviewed in 130, 131]. Of these, one CDK4/6 selective compound, palbociclib (PD-0332991, Ibrance^®^) (Figure 1), a pyrido[2,3-*d*]pyrimidine derivative, is exquisitely specific for CDK4 and CDK6, and inhibits these two kinases *in vitro* with IC_50_ values of 0.011 and 0.015μmol/L, respectively [132]. In its early stages of pre-clinical development, this compound was extensively studied for its efficacy in RB-positive tissue culture model systems as well as in mouse xenograft models of colorectal cancer (CRC), mantle cell lymphoma (MCL) and disseminated myeloma, where it induced G_1_ arrest and showed excellent efficacy [132–141]. Therapeutic doses of palbociclib resulted in a marked reduction of both phosphorylated RB and the proliferative marker Ki-67 in the tumor tissue and the downregulation of E2F-target genes. Based on these very promising results, this compound entered Phase I clinical trials in 2004, with early results indicating tolerable side effects [133–136, 142–144; reviewed in 50]. Unfortunately, the efficacy profile of this compound as a single agent was somewhat disappointing, resulting in disease stabilization rather than regression. However, results from phase II and III trials testing palbociclib in combination therapy were far more encouraging. Accelerated approval for palbociclib in combination with letrozole (Femara^®^), an aromatase inhibitor, for the treatment of advanced ER+HER2- breast cancer was granted in 2015 based on the results of the Phase II PALOMA-1 trial. Improved progression-free survival was observed with palbociclib and letrozole combination therapy compared to treatment with letrozole as a single agent in the subsequent Phase III PALOMA-2 trial. Finally, approval of palbociclib for a second indication, in combination with fulvestrant following progression on endocrine therapy, was granted based on the phase 3 PALOMA-3 study [145, 146]. Today, palbociclib is widely used in the treatment of advanced hormone receptor-positive (HR+) metastatic breast cancer (MBC) in combination with either an aromatase inhibitor or fulvestrant [reviewed in 147–149].

Owing to the success of palbociclib, additional orally bio-available CDK4 inhibitors with low nanomolar IC_50_ values against CDK4/6 have been approved for use in the treatment of HR+/HER2- metastatic breast cancers (MBCs) in combination with other approved therapies. Ribocilib (LEE011, Kisqali^®^) (Figure 1) received approval from the FDA in 2017 and inhibits CDK4 and CDK6 with IC_50_ values of 10 and 39 nM, respectively [150, 151; reviewed in 152–154]. Although effective as a single agent, like palbociclib, combination of ribociclib with an anti-estrogen is more effective in inhibiting tumor growth and RB phosphorylation [151]. Similarly, abemaciclib (LY2835219, Verzenio^®^) (Figure 1), which inhibits CDK4 and CDK6 with IC_50_ values of 2 and 5 nM, respectively, has received approval from the FDA for use in HR+/HER2- MBCs as a monotherapy for patients with progressive disease that are being treated with endocrine and chemotherapy and in combination with fulvestrant for patients whose disease has progressed following endocrine therapy [reviewed in 153].

Given the survival benefits for advanced ER+ breast cancer observed with all of the CDK4/6 inhibitors developed thus far, their use in adjuvant therapy for early stage, high-risk ER+ breast cancer was studied in several subsequent clinical trials, with somewhat conflicting results. Although a benefit with respect to improved disease-free survival was observed in the MonarchE trial [155] favoring the addition of abemaciclib for two years to standard endocrine therapy, there was no significant survival difference with the addition of palbociclib to endocrine therapy in the PALLAS study [156]. While there were slight differences in the enrolled patient populations, there may be intrinsic biological differences among the different CDK4/6 inhibitors.

The utility of CDK4/6 inhibitors as monotherapy or in combination with other targeted therapeutics is being explored in breast cancer and other tumor types. Agents that block PI3K signaling are of particular interest as the latter is activated in greater than 70% of breast cancers [157]. Pre-clinical studies using TNBC patient derived xenograft and immunocompetent syngeneic animal models have shown that combining CDK4/6 inhibitors with those that block CK1ε [158] as well PI3Kα [159] resulted cell cycle arrest, apoptosis, the activation of tumor infiltrating T-cells and an increase tumor immunogenicity. Short-term inhibition of CDK4/6 has also been shown to sensitize ER+ breast cancers to radiation therapy in pre-clinical models [160], highlighting an additional use for these agents in combination therapy.

## BIOMARKERS OF CDK4/6 INHIBITOR RESPONSE

In preclinical studies using breast cancer cell lines, an intact RB axis was required for sensitivity to CDK4/6 inhibitors. These cell lines and tumors, which express functional RB, responded remarkably well to combination therapy using CDK4/6 inhibitors with ER antagonists when compared to use of either inhibitor as a single agent as evidenced by loss of RB phosphorylation, a reliable biomarker of response to CDK4. However, functional RB may not be a requirement for response in other pre-clinical cancer models [reviewed in 147, 148]. Given the rarity of *RB* gene deletion/mutation in ER+ breast cancer, multiple genes or gene signatures may be required to accurately predict clinical sensitivity to these inhibitors. Several candidates such as *CCND1* (amplification) and p16INK4A (loss) have been studied as single biomarkers of sensitivity but have not borne out in the PALOMA-1 trial and other clinical studies. Consequently signatures aimed at identifying tumors with CYCLIN D activation, RB loss of function or CDK4 inactivation are being explored in clinical samples to distinguish those cancers that are sensitive or resistant to CDK4/6 inhibitor therapies [reviewed in 161]. For example, in the NeoRHEA Phase II trial, biopsies obtained before and after neoadjuvant treatment with palbociclib and endocrine therapy are being used to validate a CDK4 inactivation gene signature as a biomarker of insensitivity to CDK4/6 inhibition [162].

### Toxicities associated with CDK4/6 inhibitors

The common side effects of CDK4/6 inhibitors can be classified into hematological and non-hematological toxicities. Although all act by a similar mechanism, the side effect profiles are different for each agent, with neutropenia and leukopenia more frequently associated with palbociclib and ribociclib whereas diarrhea is more frequently seen with abemaciclib.

#### Hematological toxicities

Myelosuppression has emerged as a class effect of CDK4/6 inhibitors. Even though grade 3 and 4 neutropenias are very common in the published trials, the rate of infections was less frequent (25–58%) and febrile neutropenia did not exceed 2%. Moreover, the neutropenia is completely reversible without G-CSF within seven days of treatment withdrawal. This suggests that the myelosuppression from CDK4/6 inhibition is different from conventional chemotherapy-induced neutropenia. The mechanism that underlies this observation has been demonstrated *in vitro* to be due to transient cell cycle arrest in bone marrow progenitors [163]. Hence, the dosing schedule recommended for the inhibitors is cyclical, for three weeks on with one week off. Myelosuppression usually emerges 15 days after the first dose of palbociclib and ribociclib, and with abemaciclib, it emerges within the first two cycles and then less frequently in subsequent cycles. Anemia and thrombocytopenia are also commonly seen in patients treated with CDK4 inhibitors (Table 1) without increases in bleeding or transfusion requirements. Treatment guidelines have been established for blood count monitoring and dose reduction due to cytopenias for this class of drugs [164–166].

**Table 1:** Summary of clinical trials of CDK4/6 inhibitors

Treatment	Clinical trial name	Clinical trial NCT	Tumor type	Grade 3-4 side effects (>20%)	Most common side effects (>30%)
abemaciclib (monotherapy)	MONARCH-1	02102490	ABC HER2+ HER2−	leukopenia neutropenia diarrhea	leukopenia^**^ diarrhea^*^ neutropenia^*^ anemia fatigue nausea decreased appetite thrombocytopenia abdominal pain vomiting
abemaciclib+ fulvestrant	MONARCH-2	02107703	ABC HER2− HR+	neutropenia	diarrhea^*^ neutropenia nausea fatigue abdominal pain
abemaciclib+ non-steroidal aromatase inhibitor	MONARCH-3	02246621	ABC HER2− HR+	neutropenia	diarrhea^*^ neutropenia fatigue infections and infestations nausea abdominal pain
palbociclib (monotherapy)		01037790	ABC	neutropenia leukopenia lymphopenia	leukopenia^*^ neutropenia^*^ thrombocytopenia anemia lymphopenia
palbociclib+ letrozole	PALOMA-2	01942135	ABC HER2− HR+	neutropenia leukopenia	neutropenia^*^ leukopenia nausea fatigue alopecia arthralgia
palbociclib+ fulvestrant	PALOMA-3	01942135	ABC HER2− HR+	neutropenia leukopenia	neutropenia^*^ leukopenia infections nausea fatigue
ribociclib (monotherapy)		01237236	lymphomas solid tumors	neutropenia	neutropenia leukopenia thrombocytopenia fatigue nausea
ribociclib+ letrozole	MONALEESA-2	02107703	ABC HER2− HR+	neutropenia leukopenia	neutropenia leukopenia nausea infections diarrhea fatigue alopecia

^*^denotes >80%, ^**^denotes 100%. Abbreviations: ABC: advanced breast cancer; HER2: human epidermal growth factor.

#### Non-hematological toxicities

Common non-hematological toxicities associated with CDK4/6 inhibitors include fatigue, diarrhea, transaminitis, increased creatinine and Qt prolongation. Diarrhea, abdominal pain and increased creatinine were seen more frequently in clinical trials with abemaciclib. In addition to blood count monitoring, liver function and Qt assessments at baseline are recommended with these drugs. More recently, the FDA has warned that rare but severe interstitial pneumonitis can be observed with CDK4/6 inhibitor treatments [167].

### CDK4 inhibitors as modulators of immune checkpoints

The role that the immune system plays in controlling tumor growth and how tumors evade the immune system has recently been thrust into the limelight as the utility of checkpoint inhibitors in cancer therapy continues to rise. While the main function of immune checkpoints is to maintain self-tolerance [168], it is now well established that cancer cells co-opt these pathways to evade destruction by the immune system. In certain cancers, such as those of the lung, kidney and skin (melanoma), immunotherapy is considered standard of care for subsets of patients with these particular tumor types [169]. However, in breast cancer and other tumor types, overall response to checkpoint inhibitors is relatively low (<20%) when administered as single agents [170]. Interestingly, it has recently been shown that CDK4/6 inhibitors induce an anti-tumor immunogenic response by 4 primary mechanisms: (1) increased antigen presentation in the tumor itself via innate and adaptive immunity, (2) enhanced T-cell activation, (3) suppressed proliferation of immunoresponsive regulatory T cells (T_regs_) and (4) induction of a memory T cell phenotype [159, 171–176]. In an effort to expand the utility and benefits of immune checkpoint therapy to breast and other cancers, several groups have exploited the immunomodulatory properties of CDK4/6 inhibitors to enhance the efficacy of immune checkpoint inhibitors. Studies in animal models clearly demonstrate that abemaciclib and palbociclib synergize with anti-PD-1 and PD-L1 antibodies to induce tumor regression, and in some cases complete regression, in immunocompetent mouse models of lung [172] and mammary tumors [159, 160, 173, 174].

**Figure 2:**
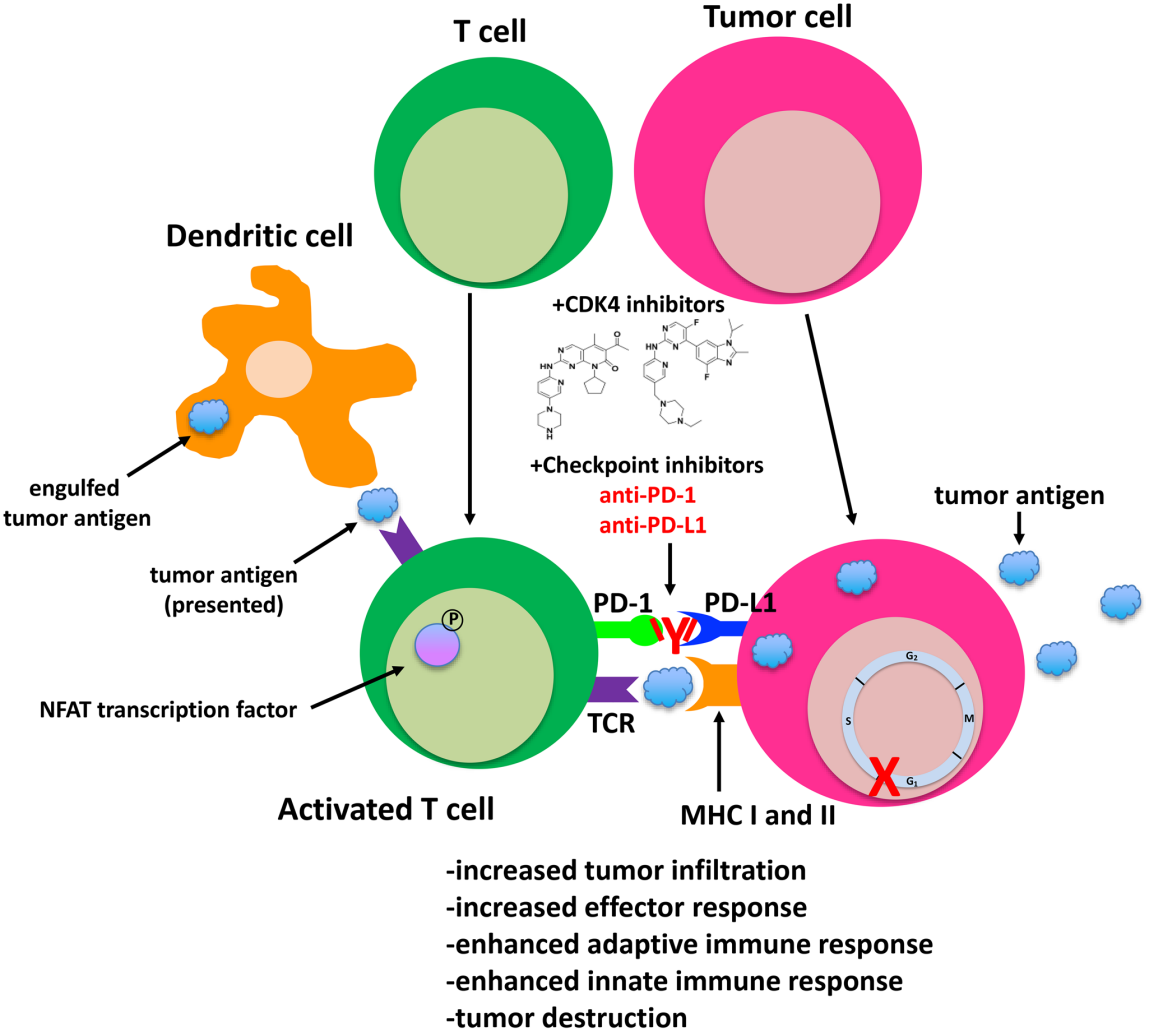
Modulation of anti-tumor and immune responses by small molecule CDK4/6 and checkpoint inhibitors.

Several mechanisms appear to underlie the synergism between checkpoint and CDK4/6 inhibitors (Figure 2). PD-L1 expression fluctuates during the cell cycle, with maximum levels observed in M phase and declining sharply thereafter [173]. Disruption of *ccnd1-3* loci and that of *cdk4* (but not *cdk6*) in MEFs, as well as treatment of tumor cell lines with CDK4 inhibitors, also stabilize PD-L1 at the protein level [173]. Inhibition of CDK4 also blocks its phosphorylation of speckle-type POZ protein (SPOP), a CULLIN-3 E3 ligase, and leads to the degradation of SPOP by FZR1, a component of the anaphase promoting complex. Because CULLIN-3^SPOP^ interacts with the PD-L1 cytoplasmic domain, PD-L1 expression is stabilized in tumors treated with CDK4 inhibitors [172]. Treatment of tumor bearing mice with CDK4/6 inhibitors has a priming effect on CD8+ T cells, leading to a memory phenotype that has a distinct gene signature and is RB-dependent [175, 176]. CDK4/6 inhibition also has a direct effect on T cell receptor (TCR) signaling by triggering nuclear localization of the nuclear factor of activated T cells (NFAT) transcription and NFAT-dependent changes in gene expression that are essential for T cell activation [172, 174]. At the genomic level, CDK4 inhibitors induce changes in the expression of genes that are associated with a T-cell-mediated inflammatory response, dendritic cell maturation and antigen presentation via downregulation of *DNMT1* [171, 174]. Accordingly, analysis of tumors treated with these inhibitors show upregulation of major histocompatibility (MHC) class I and II and antigen presenting cells [174].

In spite of these observations, early phase I/II clinical data on combination CDK4/6 inhibitor/letrozole and anti-PD-1 (pembrolizumab) treatment demonstrated increased but tolerable toxicities in the absence of improved efficacy in terms of survival [177] when compared to that observed in the earlier PALOMA-2 clinical trial. Mechanistically, no significant differences in tumor-infiltrating lymphocytes, PD-L1 levels, or gene-expression profiles that are typically associated with CDK4/6 inhibitor resistance were observed between patients who showed a complete response versus those who did not [177]. As these data represent some of the earliest trial results obtained for CDK4/6 inhibitor/immunotherapy combination treatment, additional studies will determine whether similar treatment regimens are of clinical benefit.

Paradoxically, inhibiting CDK4/6 might be a double-edged sword. Studies using a genetically modified mouse model of *Eμ-myc*-induced B-cell lymphoma showed that disruption of the *cdk4* locus leads to genomic instability and accelerated lymphoma development via FOXO1 [178]. Analysis of human non-Hodgkin B-cell lymphoma specimens showed that CDK4 protein expression is down-regulated in several sub-types, which correlated with reduced levels of *CDK4* transcripts, pointing to a tumor-suppressive role for CDK4 in MYC-driven B-lineage malignancies via suppression of genomic instability. These conclusions are supported by previous studies of CDK6, which is also targeted by palbociclib and other CDK4 small molecule inhibitors, and its role in BCR-ABL transformed B lineage cells. Kolmann et al. [179] demonstrated that overexpression of CDK6 in such cells delayed their cell cycle progression and suppressed their ability to promote leukemogenesis in mice by inducing expression of p16^INK4A^. The authors also reported that expression of CDK6 inversely correlated with that of p16^INK4A^ in human B- and T-cell lymphomas. Together, these and other studies in lymphoid cells suggest that therapeutic targeting of CDK4/6 might promote unwanted phenotypes in a cell-type dependent manner.

### Resistance to small molecule CDK4/6 inhibitors

**Figure 3:**
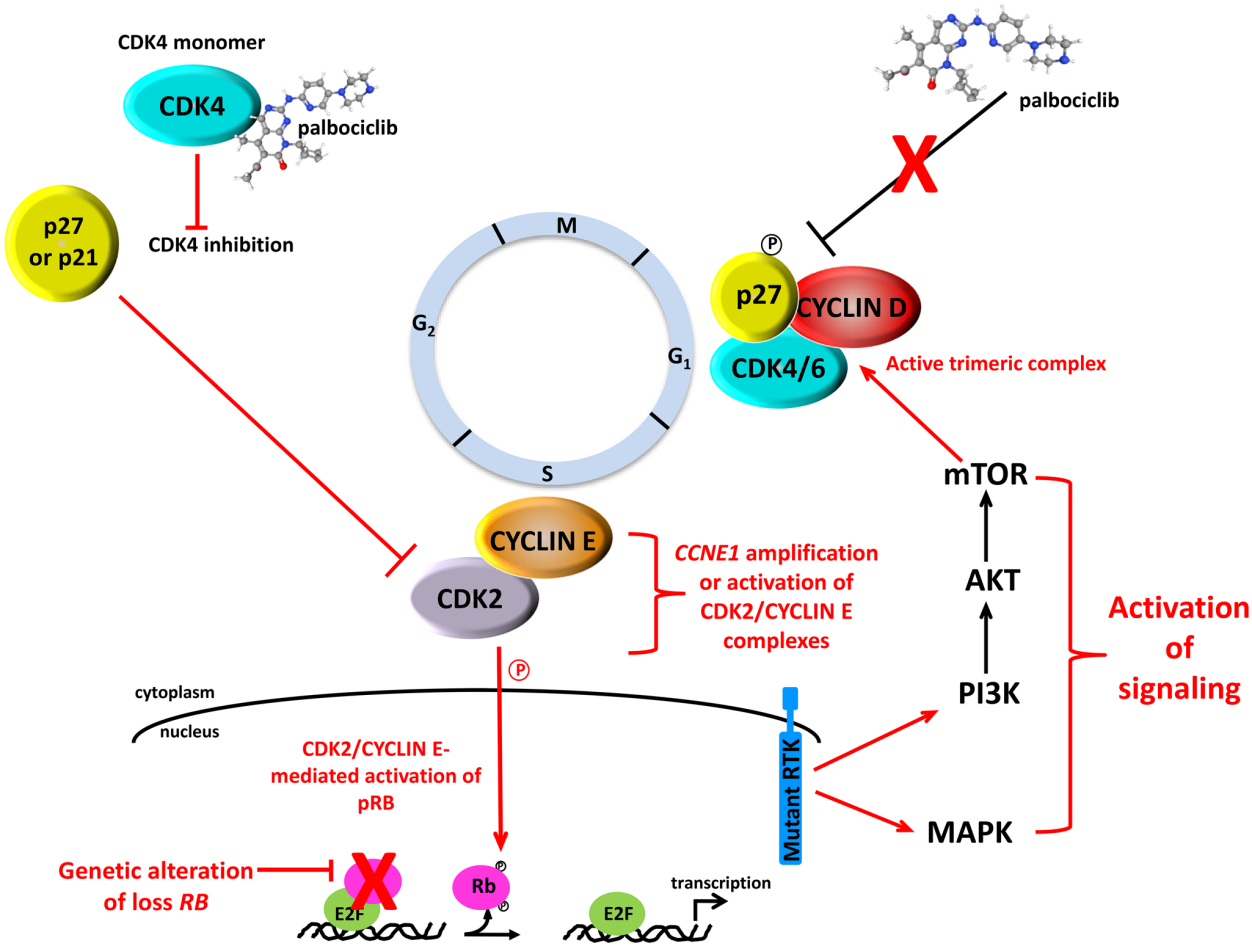
Mechanisms that underlie resistance to CDK4/6 inhibitors. (Adapted and modified from [50]).

There are several mechanisms by which tumors can acquire resistance to or simply fail to respond to CDK4/6 inhibition [reviewed in 180] (Figure 3). As previously mentioned, the earliest studies that pre-dated clinical approval of these agents in ER+ MBC demonstrated that response was largely observed in ER+ vs. ER- breast cancers, suggesting that mutations in estrogen receptor (*ESR*) genes and others that play a role in ER signaling might lead to resistance. Although mutations in the *ESR1* gene can be responsible for resistance to endocrine therapy [181–184], they do not drive resistance to CDK4/6 inhibition [185]. A recent study of MBCs with acquired resistance to ER targeted therapies revealed that mutations in *ERBB2* not only conferred estrogen-independent growth and resistance to fulvestrant and tamoxifen, but also resistance to palbociclib, suggesting that use of a HER2 inhibitor would be effective in this setting [186]. Given that an intact RB pathway is key to response, a second and more obvious cause of resistance is loss or mutation of RB as well as other genes encoding cell cycle machinery. While not an all-inclusive list, studies using established breast tumor cell line and patient-derived xenograft models as well as retrospective analysis of clinical trial data have shown that resistance to CDK4/6 inhibition is associated with downregulation of RB expression, activation of interferon (IFN) signaling, FGFR1 amplification, overexpression of *CCNE1* and overexpression/activation of CDK2/CYCLIN E1 complexes [187–189, reviewed in 190, 191].

Cells that are resistant to CDK4/6 inhibition also employ alternative mechanisms to drive proliferation and escape growth arrest [reviewed in 191, 192]. One of the initial kinome-wide studies aimed at identifying druggable pathways in CDK4/6 inhibitor-resistant cells revealed that 3-phosphoinositide-dependent kinase 1 (PDK1) was upregulated in terms of overall expression levels and phosphorylation in cells treated that acquired resistance to ribociclib [193]. Consistent with previous studies, cell cycle progression in these cell lines was driven by S-phase CYCLIN/CDK2 complexes. Both genetic and pharmacologic inhibition of PDK1 sensitized ER+ breast cancer cells to and acted synergistically with ribociclib *in vitro* and *in vivo*. Combination studies also revealed that cells treated with these inhibitors underwent apoptosis, which is in contrast to the cytostatic effects and senescence-inducing properties of CDK4/6 inhibitors [193]. The results of subsequent RNA-Seq and kinome analyses have continued to underscore reliance on the phosphatidyl-inositol-3-kinase/mammalian target of rapamycin (PI3K-mTOR) signaling axis [194] in CDK4/6 inhibitor-resistant cells, whereby mTORC1/2 inhibition in palbociclib-resistant cells reduces tumor growth [195, 196] and reactivates the CDK4/6-RB signaling node with commensurate changes in E2F-mediated transcription [195], without inducing a senescence-like phenotype. The fact that abnormal PI3K-mTOR as well as MAPK signaling are observed in multiple tumor types that exhibit *do novo* or acquired resistance to CDK4/6 inhibitors [reviewed in 191, 192] suggests that targeting these pathways might be of significant clinical value. A number of clinical trials with PI3K/mTOR-targeted therapies in combination with CDK4/6 inhibitors for use in advanced HER2+/− breast cancer are ongoing [reviewed in 191], as those that are biomarker-driven in order to assess what agents would be beneficial to patients as second-line therapeutic regimens. Phase I-III trials examining the therapeutic benefit of selective estrogen receptor degraders as well as small molecule CDK2, BCL-2 (venetoclax/Venclexta^®^), FGFR, AURKA and immune checkpoint inhibitors (discussed above) are also active and/or completed [reviewed in 197].

### Mechanism of action of CDK4/6 inhibitors

X-ray crystallography studies have revealed a surprising mechanism of action associated with palbociclib-mediated inhibition of CDK4 [198]. Dr. Rubin’s group determined the crystal structure of the CDK4 holoenzyme, which revealed that p27 binds to CDK4 and allosterically activates it by remodeling the ATP-binding site, thereby promoting release of the kinase activation segment leading to RB phosphorylation. They also found that phosphorylation of Tyr74 in p27 is essential for the activation CDK4 and that the lack of such a tyrosine residue in p21 makes it a poor activator of CDK4. As previously discussed, p27 is expressed throughout the cell cycle where it is associated with CYCLIN D-CDK4 complexes [22, 23, 25–29; reviewed in 11, 12]. However, the mechanism by which it activates these complexes was not well defined. In this context, studies by Guiley et al. [198] show that the CYCLIN D1-CDK4-p27 trimeric complex is resistant to the effects palbociclib and other approved CDK4 inhibitors. Instead, palbociclib primarily targets CDK4/6 monomer and promotes the formation of inactive CDK2-p21 complexes leading to inactivation of CDK2 enzyme activity, which is essential for sustained RB inhibition and growth arrest [198] (Figure 3). Subsequent stability studies by Pack et al. [199] using the 3 clinically approved CDK4/6 inhibitors have shown that they induce immediate dissociation of p21 from CDK4 while leaving CDK6 complexes intact. Therefore, while p21 is able to indirectly inhibit CDK2, p27 is unable to do so due to the fact that it remains bound to CDK4/CYCLIN D1 complexes [199] which are refractory to CDK4 inhibitors. As the authors of both studies study point out, inhibition of CDK2 is of paramount importance for the induction of cell cycle arrest in breast tumor cells that respond to CDK4/6 inhibitors and without CDK2 inhibition, patients will undoubtedly develop resistance to this class of therapeutics. Ongoing and future clinical trials examining the utility of CDK2 inhibitors in patients that progress while undergoing CDK4/6 inhibitor treatment will hopefully address this issue.

Despite the effectiveness of CDK4/6 inhibitors in HR+ breast cancer, their preclinical and clinical effectiveness has been limited in other predominantly RB-proficient tumor types, such as in NSCLC, colorectal cancer and melanoma [200, 201]. Closer examination revealed that expression of CDK6 affects response to CDK4/6 inhibitors. Tumors with low levels of CDK6 compared to CDK4 are universally sensitive to CDK4/6 inhibitors, including HR+ breast, mantle cell lymphomas, Ewing sarcomas as well as subsets of large tumor types, e.g. NSCLC. By contrast, when both CDK6 and CDK4 are coexpressed in a tumor at comparable levels, CDK6 drives Rb/E2F output. In the vast majority of RB-proficient solid tumors that are intrinsically resistant to CDK4/6 inhibitors, CDK6 is expressed in a thermostable conformation (CDK6-stable) with weak binding to current CDK4/6 inhibitors [202]. New inhibitors able to bind and inhibit CDK6-stable are warranted to effectively suppress Rb/E2F output in these tumors.

### CDK4/6 PROTACs

The strategy of Proteolysis Targeted Chimeras (PROTACs) has recently gained traction in the field of drug development [reviewed in 203]. PROTACs are heterobifunctional small molecules that include a ligand binding to a target protein and a moiety binding to an E3 ubiquitin ligase, conjugated by a linker aimed at degrading the target protein through the ubiquitin-proteasome system. A number of recent studies have reported on CDK4/6-directed PROTACs and their potential use as biochemical tools or potential therapeutics [204–210]. A notable sub-class of PROTACs showed significantly better degradation of CDK6 over CDK4, suggesting a potential therapeutic use in certain leukemias such as in AML and Ph+ ALL [202, 210, 211]. CDK4/6-directed PROTACs can be also used as a tool to indirectly determine target engagement in living cells. In one study, a CDK4/6 directed PROTAC revealed weak target engagement of CDK6 by CDK4/6 inhibitor in many solid tumors, indicating intrinsic resistance to CDK4/6 inhibitors. In the same solid tumor cells, a CDK6-selective PROTAC was also unable to degrade CDK6 [202], emphasizing the need for development of potent inhibitors of CDK6-stable to be used in future PROTAC development for these tumors.

Another distinguishing feature across CDK4/6 PROTACs that may potentially influence their therapeutic implications, is the ability of some but not all to induce degradation of the canonical “imid” targets, Icaros and Aiolos. Other studies are needed to determine whether this additional property will contribute increased antitumor effectiveness, as suggested by Jiang et al. [205], or if it will potentially add toxicities.

## A NON-THERAPEUTIC ROLE FOR CDK4 INHIBITORS AS PREVENTATIVE AGENTS FOR CHEMOTHERAPY-INDUCED DEPLETION OF HEMATOPOIETIC STEM CELLS

One of the unfortunate side effects of systemic chemotherapy with traditional cytotoxic agents is immunosuppression that arises due to bone marrow (BM) toxicity, with the acute effects being predominantly due to the destruction of hematopoietic stem cells and progenitors (HSPCs). The proliferation of these cell types is dependent on CDK4/6 activity due to the fact that CYCLIND1/CDK4 complexes drive the G_0_-G_1_ transition and shorten the length of the G_1_ phase [212]. In a recent study, He et al. [213] reported that treatment of mice with trilaciclib (G1T28), a clinically-approved CDK4/6 inhibitor that was developed to reduce chemotherapy-induced myelosuppression [214], transiently arrests HSCs and progenitors in the G_1_ phase of the cell cycle. Although this result was not surprising, the authors cleverly extended these studies to show that a single dose of trilaciclib 30 minutes prior to a single or repeated dose of 5-fluorouracil (5-FU) also induces a transient G_1_ arrest in the hematopoietic stem and progenitor cells (HSPCs) in mice, preserved HSC function and reduced the degree of myelosuppression due to HSC exhaustion. These effects were intrinsic to the BM and lasted long-term as demonstrated by BM transplantation assays. Interestingly, primary and secondary transplant recipients did not develop any hematological malignancies in a 1-year time period following repeated combination dosing, suggesting that trilaciclib and other CDK4/6 inhibitors might also prevent secondary myeloid neoplasms that emerge in a small percentage of patients treated with systemic chemotherapeutics that induce HSC exhaustion and myelosuppression. Further studies using syngeneic or other immunocompetent models with tumors that are not dependent on CDK4/6 activity, as well as determining whether the non-hematological AEs associated with CDK4/6 inhibitors (discussed above) are likely to shed further light on the utility of these drugs in the context of HSPC protection.

## SUMMARY

CDK4/6 is a key mediator of cell cycle progression through the G_1_ phase, the time when a cell prepares to initiate DNA synthesis. The cell’s reliance on this protein as well as its CYCLIN D binding partners and downstream target RB for proliferation underscores why the CDK4/CYCLIN D/RB signaling module is often deregulated in transformed cells. The approval of 3 CDK4/6 inhibitors as treatments for ER+ breast cancer has paved the way for ongoing clinical studies evaluating the utility of these inhibitors in combination with those of other signaling pathways (such as but not limited to BRAF, PI3K and MEK) in multiple tumor types that exhibit reliance on CYCLIN D1/CDK4/RB or other components of the cell cycle such as p16 and p27. The success of these trials, as well as understanding the mechanisms that drive resistance to these inhibitors, should provide an answer as to whether selective inhibitors of CDK4/6 can provide therapeutic benefit in a broader array of cancers.

## Abbreviations

BCSC: breast cancer stem cell
BM: bone marrow
CDK: cyclin-dependent kinase
ER: estrogen receptor
HR: hormone receptor
HSC: hematopoietic stem cell
MBC: metastatic breast cancer
MMTV: mouse mammary tumor virus
PD-1: programmed cell death protein
PD-L1: programmed death-ligand 1
PROTAC: proteolysis targeting chimera
RB: retinoblastoma
